# Patients’ Experiences of a Nurse-Guided Physical Activity Program for Type 2 Diabetes and Its Perceived Impact on Health and Quality of Life: A Qualitative Descriptive Study

**DOI:** 10.3390/healthcare14172756

**Published:** 2026-08-31

**Authors:** Jamal M. Alzahrani, Abdulaziz M. Alodhailah, Monirah Albloushi, Majed M. Aljabri, Mohammed Almutairi

**Affiliations:** 1 Exercise Physiology Department, College of Sport Sciences and Physical Activity, King Saud University, Riyadh 11451, Saudi Arabia; 2 Department of Medical-Surgical Nursing, College of Nursing, King Saud University, Riyadh 11451, Saudi Arabia; 3 Department of Community and Psychiatric Mental Health Nursing, College of Nursing, King Saud University, Riyadh 11451, Saudi Arabia

**Keywords:** type 2 diabetes mellitus, nurse-guided intervention, physical activity program, patient experiences, qualitative research, quality of life, Saudi Arabia

## Abstract

Background: Type 2 diabetes mellitus (T2DM) represents a significant global health challenge, with physical activity recognized as a cornerstone of effective disease management. Nurse-guided physical activity programs offer a promising approach to enhancing glycemic control and quality of life; however, patients’ lived experiences of such interventions remain underexplored, particularly in Middle Eastern contexts. Aim: To explore the perceived experiences of patients with T2DM participating in a nurse-guided physical activity program and understand its role in improving their health outcomes and quality of life. Methods: A qualitative descriptive design was employed. Sixteen adults with T2DM who completed a 12-week nurse-guided physical activity program were purposively recruited from multiple healthcare institutions in Saudi Arabia, including both public and private facilities. Semi-structured interviews were conducted using a 20-item interview guide. Data were analyzed using Braun and Clarke’s six-phase reflexive thematic analysis. Trustworthiness was ensured through member checking, peer debriefing, and maintaining an audit trail. The study adhered to the Consolidated Criteria for Reporting Qualitative Research (COREQ) guidelines. Results: Four overarching themes emerged: (1) Transformative Health Journey, encompassing perceived physiological improvements and enhanced energy levels; (2) Psychological Awakening, reflecting improved mood, self-efficacy, and reduced diabetes-related distress; (3) The Pivotal Role of Nursing Support, highlighting the significance of professional guidance, monitoring, and therapeutic relationships; and (4) Navigating Challenges and Charting Future Directions, addressing barriers, facilitators, and recommendations for program enhancement. Conclusions: Nurse-guided physical activity programs offer multidimensional benefits for patients with T2DM, extending beyond physiological improvements to encompass psychological well-being and lifestyle transformation. Nursing professionals play a critical role in motivating, educating, and supporting patients throughout their health journey. These findings support the integration of nurse-led physical activity interventions into routine diabetes care.

## 1. Introduction

Type 2 diabetes mellitus (T2DM) has emerged as one of the most pressing global health challenges of the 21st century, with the International Diabetes Federation (IDF) estimating that 537 million adults were living with diabetes in 2021, projected to reach 783 million by 2045 [1]. The Middle East and North Africa region bears a disproportionate burden, with Saudi Arabia ranking among the top ten countries globally for diabetes prevalence, affecting approximately 28% of the adult population [2]. This epidemic poses substantial implications for healthcare systems, economic productivity, and individual quality of life.

The management of T2DM extends beyond pharmacological interventions to encompass comprehensive lifestyle modifications, with physical activity constituting a fundamental pillar of effective disease management [3]. Regular physical activity has been demonstrated to improve glycemic control, reduce cardiovascular risk factors, enhance insulin sensitivity, and promote psychological well-being [4,5]. Despite robust evidence supporting these benefits, adherence to physical activity recommendations among individuals with T2DM remains suboptimal, with studies reporting that fewer than 40% of patients meet recommended levels of physical activity [6].

Multiple barriers contribute to this adherence gap, including lack of motivation, insufficient knowledge, time constraints, physical limitations, and inadequate professional support [7]. These challenges underscore the need for structured, professionally guided interventions that address both the physical and psychosocial dimensions of physical activity promotion. Within this context, nurse-guided physical activity programs have emerged as a promising approach, leveraging nurses’ unique position at the interface of patient education, health promotion, and chronic disease management [8].

Nurses, as the largest healthcare workforce globally, possess distinctive competencies in patient-centered care, health education, and therapeutic relationship building that position them ideally to design and implement physical activity interventions [9]. Nurse-guided programs offer advantages including accessibility, continuity of care, holistic assessment, and the ability to tailor interventions to individual patient needs and preferences [10]. However, while quantitative studies have documented the effectiveness of such programs in improving clinical outcomes, there remains a paucity of research exploring patients’ lived experiences, perceptions, and the meanings they ascribe to their participation in nurse-guided physical activity interventions.

Understanding patients’ experiences is essential for several reasons. First, qualitative insights can illuminate the mechanisms through which interventions exert their effects, informing theoretical understanding and program refinement [11]. Second, patient perspectives can identify previously unrecognized barriers and facilitators, enabling the development of more patient-centered interventions [12]. Third, exploring the subjective dimensions of physical activity participation, which encompass psychological, social, and existential meanings, provides a more comprehensive understanding of the intervention impact beyond objective clinical parameters [13].

In the Saudi Arabian context, cultural, social, and environmental factors may shape patients’ experiences with physical activity programs in distinctive ways. These include gender-specific considerations, family dynamics, religious practices, climatic conditions, and healthcare system structures [14]. To date, limited qualitative research has explored how Saudi patients with T2DM experience and perceive nurse-guided physical activity interventions, representing a significant gap in the literature.

### 1.1. The Global Burden of Type 2 Diabetes Mellitus

T2DM is a chronic metabolic disorder characterized by insulin resistance and progressive beta-cell dysfunction, resulting in hyperglycemia and associated complications [15]. The condition is associated with significant morbidity, including cardiovascular disease, nephropathy, retinopathy, neuropathy, and increased mortality risk [16]. Beyond physical health, T2DM substantially impacts psychological well-being, with elevated rates of depression, anxiety, and diabetes-related distress documented across populations [17].

The economic burden of diabetes is equally substantial, with global healthcare expenditure attributable to diabetes estimated at $966 billion in 2021 [1]. In Saudi Arabia, diabetes-related healthcare costs represent a significant proportion of the national health budget, with projections indicating continued escalation without effective prevention and management strategies [18].

### 1.2. Physical Activity as a Cornerstone of Diabetes Management

Physical activity encompasses any bodily movement produced by skeletal muscles that requires energy expenditure, including structured exercise, occupational activity, and activities of daily living [9]. For individuals with T2DM, regular physical activity confers multiple benefits through distinct physiological mechanisms. Acute effects include enhanced glucose uptake through insulin-independent pathways, whereas chronic adaptations encompass improved insulin sensitivity, favorable body composition changes, and cardiovascular conditioning [19].

Current guidelines recommend that adults with T2DM engage in at least 150 min per week of moderate-intensity aerobic activity, supplemented by resistance training on two or more days per week [3]. However, the translation of these recommendations into sustained behavioral change represents a persistent challenge. Factors contributing to low adherence include knowledge deficits regarding appropriate activities, fear of hypoglycemia, physical limitations, competing time demands, and insufficient healthcare provider guidance [20].

### 1.3. The Role of Nursing in Physical Activity Promotion

Nurses occupy a strategic position in promoting physical activity among individuals with chronic conditions. The nursing profession’s philosophical foundations, which emphasize holistic care, patient empowerment, and health promotion, align intrinsically with physical activity counseling [21]. Moreover, nurses’ frequent patient contact, established therapeutic relationships, and expertise in patient education create opportunities for sustained engagement and support.

Nurse-led interventions for physical activity promotion have demonstrated effectiveness across various chronic conditions. Systematic reviews have reported significant improvements in physical activity levels, self-efficacy, and clinical outcomes following nurse-delivered interventions [22,23]. For T2DM specifically, nurse-guided programs have shown promise in improving glycemic control, reducing cardiovascular risk factors, and enhancing quality of life [24].

Despite these positive findings, the literature remains predominantly quantitative, focusing on measurable outcomes while providing limited insight into patients’ subjective experiences. Qualitative research offers methodological tools to explore these experiential dimensions, generating rich, contextualized understanding that can inform intervention development and implementation [25].

### 1.4. The Saudi Arabian Context

Saudi Arabia presents a unique context for diabetes management research. The nation has experienced rapid socioeconomic transformation over recent decades, accompanied by shifts in lifestyle patterns, including reduced physical activity and dietary changes that have contributed to escalating diabetes prevalence [14]. Cultural factors, including gender-specific activity spaces, family-centered decision-making, and religious considerations, may influence patients’ engagement with physical activity programs in ways that differ from Western contexts [26].

The Saudi healthcare system has increasingly emphasized primary care strengthening and chronic disease management through Vision 2030 reforms, creating opportunities for expanded nurse-led initiatives [27]. However, research exploring Saudi patients’ experiences with such programs remains scarce, limiting evidence-based program development and refinement.

This study aimed to explore the perceived experiences of patients with type 2 diabetes mellitus who participated in a nurse-guided physical activity program and to understand the program’s role in improving their health outcomes and quality of life. Specifically, the study sought to (1) explore participants’ overall experiences with the nurse-guided physical activity program; (2) understand perceived changes in physiological indicators and general health status; (3) examine the impact on psychological well-being and quality of life; (4) identify factors facilitating and hindering program participation and adherence; (5) describe participants’ perceptions of nursing support and guidance; and (6) generate recommendations for program improvement from participants’ perspectives.

## 2. Materials and Methods

### 2.1. Study Design and Setting

A qualitative descriptive design was employed to explore participants’ experiences with the nurse-guided physical activity program. Qualitative description, as articulated by Sandelowski [28,29], represents a legitimate and valuable approach when the research goal is to provide a comprehensive summary of events in everyday terms. This design is particularly appropriate when researchers seek to describe phenomena from participants’ perspectives without imposing a priori theoretical frameworks or generating highly abstract interpretations [30].

The qualitative descriptive approach was selected for several reasons. First, it enables researchers to remain close to participants’ words and meanings while providing systematically analyzed findings [31]. Second, it offers flexibility in sampling, data collection, and analysis procedures while maintaining methodological rigor [28]. Third, it generates findings with direct relevance for clinical practice and program development, aligning with the study’s applied aims [32].

This study adhered to the Consolidated Criteria for Reporting Qualitative Research (COREQ) guidelines to ensure comprehensive and transparent reporting [33]. The complete COREQ checklist is provided in the Supplementary Materials (Supplementary File S1).

This study was guided by a naturalistic paradigm, acknowledging multiple, constructed realities shaped by individuals’ experiences and social contexts [34]. Within this paradigm, knowledge is understood as co-created through the interaction between researcher and participant, with findings representing contextually bounded interpretations rather than universal truths [35].

Epistemologically, the study adopted a subtle realist position, recognizing an external reality while acknowledging that access to this reality is mediated by human perception and interpretation [36]. This position allows for descriptive findings that reflect participants’ genuine experiences while recognizing the researcher’s role in constructing the research account.

The study was conducted among participants recruited from multiple healthcare institutions in Saudi Arabia, including both public and private facilities. These institutions provide comprehensive diabetes management services, including medical care, patient education, and lifestyle counseling. The nurse-guided physical activity program was implemented as part of an integrated diabetes care approach, conducted over 12 weeks with sessions held three times weekly. No formal comparison of participants’ experiences or perceptions by healthcare institution type (public versus private) was undertaken in this study.

The program involved structured aerobic exercises (including walking, stationary cycling, and low-impact aerobics) and resistance training activities tailored to participants’ physical capabilities. Each session lasted 45–60 min and was supervised by trained nursing staff who provided guidance, motivation, monitoring of physiological responses, and individualized feedback. Nurses also delivered education regarding the benefits of physical activity, self-monitoring techniques, and strategies for integrating activity into daily routines. Before delivering the intervention, nurses received standardized training covering the program protocol, exercise procedures, participant safety, monitoring of physiological responses, motivational support, self-monitoring techniques, and delivery of physical activity education. Sessions were conducted according to a standardized intervention schedule, with session times arranged according to participants’ availability. Each participant was supervised by the same nurse throughout the 12-week program to promote continuity of care and consistency in intervention delivery. The intervention was implemented using a standardized written protocol and structured session procedures to ensure consistency across sessions. Adherence to supervised sessions was tracked via attendance records maintained by program staff, yielding the session attendance percentages reported for each participant (range 78–100%; Table 1); no participant’s attendance fell low enough to trigger exclusion or discontinuation.

**Figure 1:**
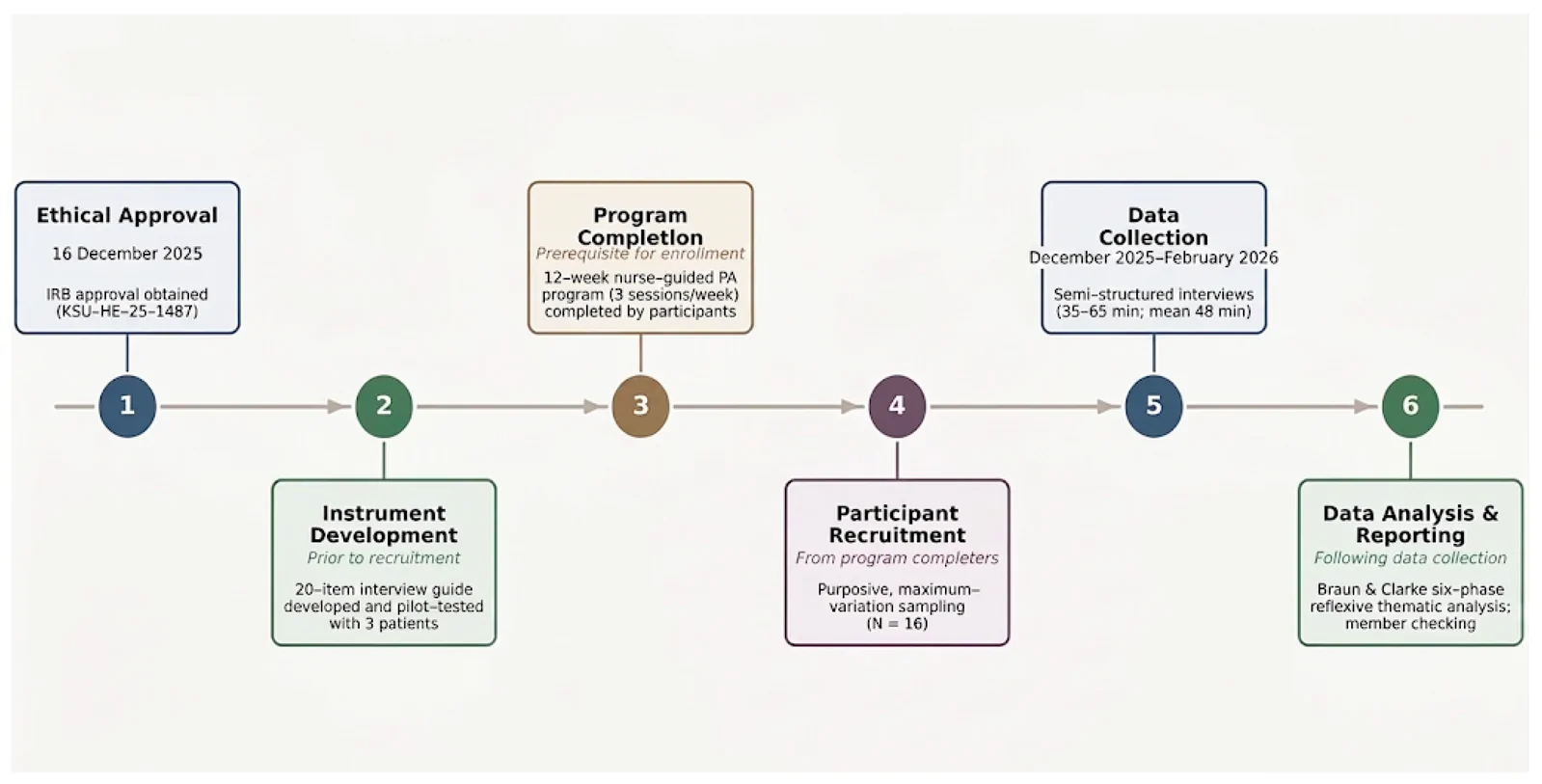
Overview of the study timeline from ethical approval to data analysis and reporting. Numbered stages indicate sequential procedural steps; dates and durations reflect the timing of the qualitative interview study.

Figure 1 provides a schematic overview of the overall study procedure, from ethical approval through data analysis and reporting.

### 2.2. Participants and Sample

Purposive sampling was employed to recruit participants who could provide rich, relevant information about their experiences with the program [37]. Purposive sampling was chosen, consistent with prior qualitative research on chronic disease self-management programs [37], because it allows deliberate selection of information-rich cases capable of speaking directly to the study aim, rather than a representative or random sample. Inclusion criteria were: (a) adults aged 18–65 years with a confirmed diagnosis of T2DM; (b) completion of the 12-week nurse-guided physical activity program; (c) ability to perform mild-to-moderate physical activity without medical contraindications; (d) willingness to share experiences through interview; and (e) ability to communicate in Arabic.

Exclusion criteria included: (a) presence of severe diabetes complications precluding safe physical activity; (b) cognitive impairment affecting ability to provide informed consent or participate in interviews; (c) concurrent participation in other structured lifestyle intervention programs; and (d) pregnancy.

Sample size was determined by the principle of information power, considering study aim, sample specificity, theoretical framework, quality of dialogue, and analysis strategy [38]. Based on published qualitative research with similar designs and populations, an initial target of 15–20 participants was established, with the final sample size determined by data saturation [39]. Data saturation was assessed iteratively throughout data collection, with recruitment continuing until no new themes emerged and existing themes were fully developed. Saturation was achieved at 16 participants.

Maximum variation sampling was employed within the purposive approach to ensure diversity across demographic characteristics (age, gender, diabetes duration, education level) and program-related factors (adherence level, perceived benefit), thereby capturing a range of perspectives [40].

### 2.3. Data Collection Instrument

Data were collected using an interview guide comprising 20 open-ended questions, developed specifically for this study and informed by the study objectives, relevant literature, and the clinical expertise of the research team (see Supplementary Materials File S2). The guide addressed key domains, including overall program experience; perceived health changes; impact on daily life and quality of life; nursing support and guidance; facilitators and barriers; and recommendations for improvement. Questions were formulated to be open and non-leading, with probing questions to encourage elaboration and clarification.

The interview guide underwent pilot testing with three patients who met inclusion criteria but were not included in the final sample. Based on pilot feedback, minor modifications were made to question wording to enhance clarity and cultural appropriateness.

### 2.4. Data Collection Procedures

Data were collected through individual semi-structured interviews conducted between December 2025 and February 2026. Semi-structured interviews were selected as the primary data collection method because they enable focused exploration of research topics while allowing flexibility to pursue emergent issues and participants’ unique perspectives [41].

Interviews were conducted by the first and second authors, members of the research team who were trained in qualitative interviewing. No pre-existing personal or professional relationship existed between the interviewers and participants prior to the study; participants were recruited from among program completers through the participating healthcare institutions, and the interviewers had no prior contact with participants outside the recruitment process. Interviews took place in private, quiet rooms at the healthcare centers at times convenient for participants. Interview duration ranged from 35 to 65 min (mean = 48 min). All interviews were audio-recorded with participants’ consent and transcribed verbatim.

Field notes were maintained throughout data collection, documenting observations regarding participant demeanor, interview context, and initial analytic reflections. These notes supplemented transcript data and informed subsequent analysis. Interviews were conducted and transcribed in Arabic, the participants’ native language. For reporting purposes, illustrative quotations included in the manuscript were translated into English by a bilingual member of the research team using a meaning-based, rather than strictly literal, approach to preserve their intended meaning, idiomatic expressions, and emotional tone. The English translations were independently checked against the original Arabic transcripts by the first author, who was also a bilingual member of the research team, to verify accuracy and consistency with participants’ original accounts.

### 2.5. Data Analysis

Data were analyzed using Braun and Clarke’s [42,43] six-phase reflexive thematic analysis. This approach was selected for its theoretical flexibility, systematic procedures, and accessibility, making it well-suited to qualitative descriptive studies seeking to identify patterns across participant accounts [44]. The six phases were implemented as follows.

- Phase 1: Familiarization with the data.

All team members independently read and re-read transcripts, listening to audio recordings to ensure accuracy and to enhance immersion. Initial observations and potential patterns were noted.

- Phase 2: Generating initial codes.

Transcripts were systematically coded using NVivo 14 software (QSR International). Both inductive codes (derived directly from the data) and deductive codes (informed by the interview guide domains and research objectives) were generated. Coding was primarily conducted by A.M.A. Consistent with the interpretive, non-consensus-based epistemology of reflexive thematic analysis [43,44], four of the sixteen transcripts were also independently read and coded by a second team member (J.M.A.). This step was intended to prompt reflexive dialogue and challenge individual interpretations rather than to establish inter-rater reliability; codes from the two researchers were therefore not reconciled into a single consensus coding frame.

- Phase 3: Generating themes.

Codes were organized into potential themes through an iterative process of comparison, combination, and refinement. Visual mapping and team discussions facilitated theme development.

- Phase 4: Reviewing themes.

Candidate themes were reviewed against coded data extracts and the entire dataset to ensure coherent patterns. Some themes were combined, others split, and all were refined to capture meaningful patterns.

- Phase 5: Defining and naming themes.

Each theme was clearly defined, specifying its scope and distinguishing features. Theme names were selected to be concise, informative, and evocative.

- Phase 6: Producing the report.

The final analysis was written, selecting vivid, representative quotations and situating findings within the broader research context.

### 2.6. Researcher Reflexivity

Reflexivity, the process of critical self-reflection regarding researchers’ backgrounds, assumptions, and positions, is essential for rigor in qualitative research [45]. The research team comprised nursing academics with clinical backgrounds in diabetes care and health promotion.

The research team brought both insider and outsider perspectives to the study. Clinical expertise in nursing and chronic disease management facilitated rapport building with participants and informed probing during interviews. At the same time, this professional background carried the potential for presuppositions that could influence data collection and interpretation. To address this, reflexive practices were embedded throughout the research process, including the maintenance of a reflexive journal documenting pre-understandings, emotional responses, and analytic decisions. Regular team discussions were conducted to critically examine how researchers’ positionalities might shape the research process and interpretations.

The research team acknowledged a generally positive orientation toward physical activity programs, consistent with their professional backgrounds in healthcare. To counterbalance this perspective, deliberate attention was given to eliciting and examining negative experiences, perceived barriers, and critical viewpoints during interviews and to ensuring these perspectives were fully and transparently represented in the analysis and reporting.

### 2.7. Trustworthiness

Multiple strategies were employed to enhance trustworthiness, guided by Lincoln and Guba’s criteria of credibility, transferability, dependability, and confirmability.

Credibility was enhanced through prolonged engagement with data; member checking, whereby summary findings were shared with five participants for verification and feedback; peer debriefing involving regular team discussions to challenge interpretations; and triangulation through multiple researchers contributing to analysis.

Transferability was supported through thick description of context, participants, and findings; purposive maximum variation sampling; and clear reporting of methods and procedures to enable readers to assess applicability to other contexts.

Dependability was achieved through maintaining a detailed audit trail documenting all methodological and analytic decisions, use of consistent procedures across interviews, and clear documentation of any protocol modifications.

Confirmability was supported through reflexive journaling, linking interpretations to data through extensive quotations, and team-based analysis processes that enabled the challenge of individual interpretations.

### 2.8. Ethical Considerations

Ethical approval was obtained from the Standing Committee for Research Ethics at King Saud University (Reference No: KSU-HE-25-1487, approved 16 December 2025). The study was conducted in accordance with the Declaration of Helsinki and Saudi Arabian research regulations.

All participants received detailed information about the study’s purpose, procedures, and their rights prior to participation. Written informed consent was obtained from each participant, emphasizing the voluntary nature of participation and the right to withdraw at any time without consequences for their healthcare. Participants were assured of confidentiality, with all identifying information removed from transcripts and replaced with pseudonyms.

Audio recordings and transcripts were stored securely on password-protected devices accessible only to the research team. No participants withdrew from the study, and no adverse events were reported. Participants were provided with contact information for support services in the event that interview discussions prompted distress, although this was not required.

## 3. Results

### 3.1. Participant Characteristics

Sixteen participants (8 males and 8 females) completed the interviews. Ages ranged from 34 to 62 years (mean = 49.3 years), and duration of diabetes ranged from 2 to 18 years (mean = 8.0 years). Educational attainment varied from secondary school to postgraduate education. All participants completed the 12-week program, with session attendance ranging from 78% to 100%. Table 1 presents detailed participant characteristics. Standard deviations for age and diabetes duration were not reported because participant-level data were not available for calculation; range and mean values were retained as the available summary statistics.

### 3.2. Overview of Themes

Four overarching themes emerged from the analysis, each comprising multiple subthemes. These themes collectively capture participants’ multidimensional experiences with the nurse-guided physical activity program. Table 2 summarizes the identified themes and subthemes with brief descriptions.

**Figure 2:**
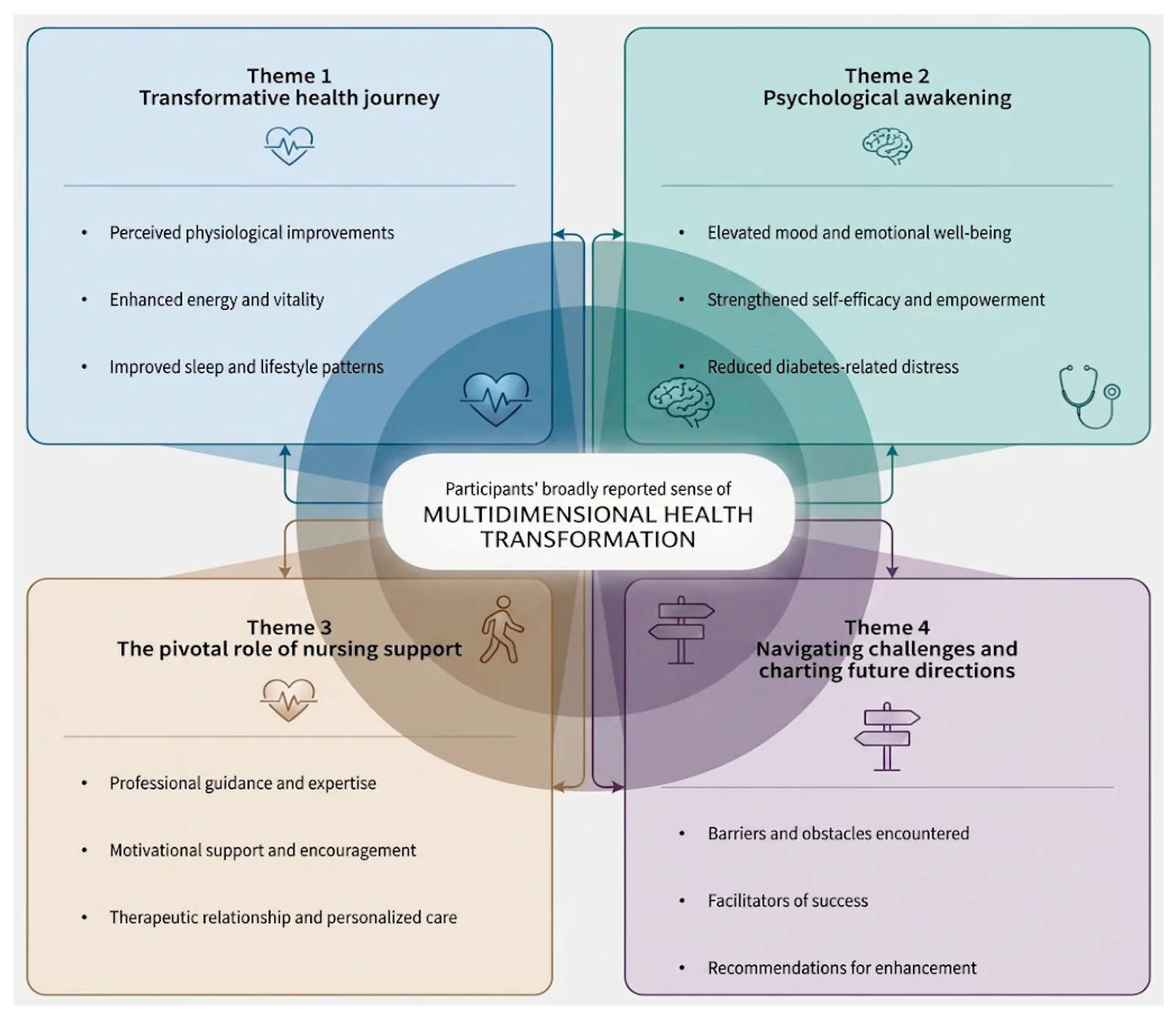
Four-quadrant matrix framework summarizing the four major themes, Transformative Health Journey, Psychological Awakening, The Pivotal Role of Nursing Support, and Navigating Challenges and Charting Future Directions, identified from participants’ accounts of the 12-week nurse-guided physical activity program. Each quadrant represents one theme; together they depict the interrelated physiological, psychological, relational, and contextual dimensions of participants’ experiences. Together, these dimensions reflect participants’ broadly reported sense of multidimensional health transformation over the course of the program.

The four key qualitative themes identified from the 12-week nurse-guided physical activity program (physiological gains, psychological benefits, nursing support, and barriers and strategies) are summarized in a four-quadrant matrix (Figure 2).

### 3.3. Theme 1: Transformative Health Journey

The first theme encapsulates participants’ perceptions of the physical health transformations they experienced through program participation. Nearly all reported positive changes in their health status, ranging from objective improvements in clinical indicators to subjective increases in energy and vitality.

#### 3.3.1. Perceived Physiological Improvements

Participants consistently described improvements in physiological indicators, including blood glucose levels, blood pressure, and body weight. These perceptions were often supported by clinical measurements, reinforcing their credibility and strengthening the participants’ commitment to continued activity.

Participant (P1), who had struggled with glycemic control, described:

*“Before the program, my fasting blood sugar was always above 200, sometimes 250. After starting the exercises with the nurses, the numbers came down to around 140 to 160, and my doctor reduced one of my medications”*.

Similarly, Participant (P2) noted changes in blood pressure:

*“I’ve had high blood pressure for years and was taking two medications. After twelve weeks of exercise, my last reading was 125 over 82, the best in years. My doctor is considering reducing my medication”*.

Several participants emphasized the motivational impact of tangible evidence. Participant (P3) explained:

*“When you see the numbers improving and the scale going down, it proves what you’re doing is working. It’s real, measurable change”*.

Weight loss was frequently mentioned. Participant (P4) shared:

*“I lost 7 kilograms. I feel lighter, move more easily, and my clothes fit better. My husband said I look healthier, which meant a lot”*.

#### 3.3.2. Enhanced Energy and Vitality

Beyond clinical measures, participants described increased energy and vitality. Many were surprised that exercise enhanced rather than depleted their energy.

Participant (P5), a retiree, reflected:

*“I thought exercise would make me more tired at my age, but it was the opposite. I now have more energy to play with my grandchildren and help at home”*.

Participant (P6) echoed:

*“Before, I came home exhausted. Now I still have energy to cook, help with homework, and spend time with my children”*.

Improved energy enhanced daily functioning. Participant (P7) noted:

*“Climbing stairs, walking to the mosque, and carrying groceries are much easier now. I don’t get breathless like before. I feel stronger and more capable”*.

#### 3.3.3. Improved Sleep and Lifestyle Patterns

Participants frequently reported better sleep and broader lifestyle improvements. Physical activity appeared to trigger positive changes in other health behaviors.

Participant (P8) described:

*“I used to have insomnia and lie awake for hours. Since starting the program, I fall asleep faster, sleep deeply, and wake up refreshed”*.

Participant (P9) highlighted dietary changes:

*“Exercise changed how I see food. I eat more vegetables, less rice, and fewer sweets. The nurses’ nutrition guidance helped a lot”*.

Several participants reported more structured routines. Participant (P10) shared:

*“The program gave structure to my days. I wake up earlier, plan meals better, and feel more organized. Exercise became a foundation for other healthy habits”*.

### 3.4. Theme 2: Psychological Awakening

This theme captures the profound psychological benefits participants attributed to the program, including improved mood, enhanced self-efficacy, reduced distress, and a transformed relationship with diabetes.

#### 3.4.1. Elevated Mood and Emotional Well-Being

Participants described meaningful improvements in mood and emotional well-being.

Participant (P11), a professional, stated:

*“Before, I was irritable and stressed. After a few weeks, my wife noticed I was calmer and more patient. I felt happier, like a cloud had lifted”*.

Participant (P12) described relief from persistent low mood:

*“Since my diagnosis, I carried a constant heaviness. After each session, I felt emotionally lighter. I looked forward to exercise as my time to release stress”*.

Group exercise enhanced emotional benefits. Participant (P13) reflected:

*“Meeting others with diabetes, laughing and encouraging each other, was healing. I made friends I still meet for walks”*.

Participant (P14) emphasized emotional release:

*“When I exercise, I forget my worries. I’m not thinking about blood sugar or complications. The stress melts away”*.

#### 3.4.2. Strengthened Self-Efficacy and Empowerment

Participants consistently reported enhanced self-efficacy and empowerment in managing diabetes.

Participant (P15) explained:

*“Before, I felt controlled by diabetes. Through this program, I realized I have power over my health. I’m not a passive patient anymore, I’m an active participant”*.

Participant (P16) shared:

*“I was afraid of exercise and hypoglycemia. The nurses taught me how to exercise safely. Now I feel confident and even started jogging on my own”*.

Empowerment extended to broader self-management. Participant (P1) noted:

*“The confidence from exercise spilled into other areas. I check my blood sugar more regularly and attend appointments more proactively”*.

Participant (P4) described a shift in identity:

*“I no longer see myself as a diabetic patient. I see myself as a healthy, active person who has diabetes. It doesn’t define me anymore”*.

#### 3.4.3. Reduced Diabetes-Related Distress

Participants reported decreased diabetes-related distress, including reduced fear and anxiety about complications.

Participant (P3) stated:

*“I used to worry about blindness and complications. The nurse showed me how exercise reduces risks. For the first time in years, I feel hopeful”*.

Participant (P6) explained:

*“Instead of feeling anxious all day, I now channel that energy into walking and exercise”*.

Participant (P5) reflected:

*“I had become bitter about my diabetes. Meeting others who were thriving changed my perspective. I realized I can live well with it”*.

### 3.5. Theme 3: The Pivotal Role of Nursing Support

Participants unanimously emphasized that nursing support was central to their engagement, persistence, and success.

#### 3.5.1. Professional Guidance and Expertise

Participants valued nurses’ clinical knowledge and expertise.

Participant (P2) stated:

*“The nurses taught proper techniques, adjusted intensity, and explained how exercise controls blood sugar. This gave me confidence”*.

Participant (P7) highlighted monitoring:

*“They checked my blood pressure and blood sugar and knew what to do when my sugar dropped. This made me feel safe”*.

Participant (P9) appreciated scientific explanations:

*“They explained how muscles use glucose and how exercise improves insulin sensitivity. Understanding the science motivated me”*.

Participant (P8) described personalized planning:

*“They created exercise plans based on my health status. This tailored approach made a real difference”*.

#### 3.5.2. Motivational Support and Encouragement

Nurses’ encouragement was described as essential.

Participant (P10) shared:

*“Knowing the nurse expected me kept me accountable. She praised my effort, which meant everything”*.

Participant (P11) stated:

*“They celebrated every small victory. Their praise motivated me to keep improving”*.

Participant (P12) reflected:

*“The difference was having someone who believed in me and reminded me of my progress when I doubted myself”*.

Participant (P13) noted:

*“The nurses set small goals and reminded me how far I’d come. Their support carried me through”*.

#### 3.5.3. Therapeutic Relationship and Personalized Care

Participants described trusting, respectful therapeutic relationships.

Participant (P14) said:

*“They knew our names and asked about our families. When I was stressed, the nurse adjusted my plan and listened. That holistic care made a difference”*.

Participant (P15) highlighted individualized care:

*“They adapted exercises for my knee problem. They treated us as individuals, not as a group”*.

Participant (P16) stated:

*“I trusted my nurse with my fears. She listened without judgment. That relationship was as valuable as the exercise”*.

Participant (P1) added:

*“They became health mentors, advising on nutrition, stress, and medication. That trusted relationship transformed how I approach my health”*.

### 3.6. Theme 4: Navigating Challenges and Charting Future Directions

This theme encompasses challenges, facilitators of success, and recommendations for future refinement.

#### 3.6.1. Barriers and Obstacles Encountered

Time constraints were the most common barrier. Participant (P4) explained:

*“Balancing work, family, and sessions was challenging. Some weeks, other obligations took priority”*.

Participant (P3) shared:

*“Work meetings conflicted with sessions. I had to negotiate flexibility with my supervisor”*.

Physical limitations also posed challenges. Participant (P5) noted:

*“With arthritis and back problems, some exercises were painful. Some days joint pain kept me home”*.

Participant (P10) stated:

*“Neuropathy made some exercises uncomfortable. Finding the right balance was challenging”*.

Environmental and cultural factors were mentioned. Participant (P6) observed:

*“The extreme heat in Riyadh makes outdoor exercise difficult. Indoor facilities were essential”*.

Participant (P2) added:

*“As a woman, appropriate exercise spaces are limited. Female-only sessions were important”*.

Family attitudes also affected participation. Participant (P14) shared:

*“Some family members didn’t understand why I prioritized exercise, which made me feel guilty”*.

#### 3.6.2. Facilitators of Success

Social support was a major facilitator. Participant (P9) stated:

*“My wife supported me and adjusted her schedule. That made everything easier”*.

Participant (P12) described peer support:

*“We formed a WhatsApp group to encourage each other. Seeing my exercise friends motivated me”*.

The program structure also helped. Participant (P11) noted:

*“Scheduled sessions made exercise an appointment I had to keep. The structure removed excuses”*.

Visible progress reinforced commitment. Participant (P15) explained:

*“Seeing improvements in blood glucose, weight, and blood pressure fueled my motivation”*.

Goal setting was beneficial. Participant (P16) shared:

*“I set goals for weight loss, walking distance, and HbA1c. The nurses helped break them into steps”*.

Intrinsic enjoyment developed over time. Participant (P7) reflected:

*“At first exercise felt like medicine. Later, I enjoyed the movement and sense of accomplishment”*.

#### 3.6.3. Recommendations for Enhancement

Participants offered practical recommendations. Participant (P3) suggested:

*“Twelve weeks starts habits, but six months or structured follow-up would help maintain them”*.

Participant (P8) recommended family involvement:

*“Including family in education would create a more supportive home environment”*.

Participant (P4) requested expanded timing:

*“Early morning or evening sessions would help working people”*.

Participant (P11) proposed technology integration:

*“An app could track progress, provide home exercises, and maintain contact with nurses”*.

Participant (P12) emphasized community building:

*“Alumni groups and peer mentorship would extend the program’s impact”*.

Participant (P2) recommended cultural considerations:

*“Continuing gender-segregated options is important, especially for Saudi women”*.

Participant (P13) concluded:

*“To make the program sustainable, we need support for independent exercise and connections to community resources”*.

Overall, the findings reveal a multidimensional transformation experienced by participants, encompassing significant physiological improvements, enhanced energy and sleep patterns, strengthened psychological well-being, and reduced diabetes-related distress. Central to this transformation was the pivotal role of nursing support, which combined clinical expertise, motivational encouragement, and therapeutic relationships to foster sustained engagement and empowerment. Although participants encountered practical, physical, and sociocultural challenges, multiple facilitators, including social support, structured programming, visible progress, and goal setting, enabled successful adherence. Collectively, these themes highlight that the program functioned not merely as an exercise intervention but as a holistic, nurse-led model of diabetes care that supported behavioral change, identity reconstruction, and long-term self-management capacity.

## 4. Discussion

This qualitative study explored the experiences of patients with T2DM who participated in a nurse-guided physical activity program, revealing multidimensional benefits and providing rich insights into the mechanisms through which such interventions influence health outcomes and quality of life. The findings advance understanding of patient-centered perspectives on nurse-led lifestyle interventions and offer practical implications for clinical practice, program development, and healthcare policy.

### 4.1. Theoretical Integration and Contribution to Knowledge

The findings align with and extend several theoretical frameworks relevant to chronic disease management and health behavior change. Participants’ accounts resonate strongly with Bandura’s Social Cognitive Theory [46], particularly regarding the concept of self-efficacy. Participants consistently described transformations in their perceived capability to manage their diabetes and engage in physical activity, changes that emerged through mastery experiences (successfully completing exercises), vicarious learning (observing peers), verbal persuasion (nurses’ encouragement), and physiological feedback (improved health indicators).

The centrality of nursing support in participants’ experiences underscores the theoretical importance of social support and therapeutic relationships in health behavior change. This aligns with Pender’s Health Promotion Model [47], which positions interpersonal influences (encompassing support, modeling, and norms) as key determinants of health-promoting behaviors. Our findings suggest that nurses provided not only informational support (education and guidance) but also emotional support (encouragement and empathy), appraisal support (feedback on progress) and instrumental support (practical facilitation of exercise participation).

The psychological benefits described by participants, specifically improved mood, reduced distress, and enhanced well-being, resonate with research on the mental health effects of physical activity [48,49]. However, our qualitative findings extend quantitative evidence by illuminating the subjective meanings participants ascribed to these changes and the mechanisms through which they perceived psychological benefits to emerge. The “psychological awakening” described by participants involved not merely mood improvement but fundamental shifts in self-perception, illness representations, and relationship with their chronic condition.

The concept of empowerment that emerged prominently in our findings connects with the theoretical construct of patient activation, defined as the knowledge, skills, and confidence needed for self-management [50]. Participants described transitioning from passive patients to active agents in their health journey, a transformation facilitated by the combination of physical activity engagement and nursing support.

### 4.2. Comparison with Existing Literature

Our findings regarding physiological improvements align with extensive quantitative evidence supporting the efficacy of physical activity interventions for T2DM [4,5]. What our study adds is insight into how patients experience and perceive these improvements. Participants described not only observing changes in clinical parameters but also integrating these observations into narratives of personal transformation and health empowerment.

The psychological benefits reported by participants are consistent with systematic reviews documenting reduced depressive symptoms, anxiety, and diabetes-related distress following physical activity interventions [51,52]. Our findings illuminate the experiential dimensions of these benefits, revealing that exercise provided participants with stress relief, hope, social connection, and a positive focus that shifted attention from disease burden to active health promotion.

The pivotal role of nursing support identified in our study corroborates research highlighting the effectiveness of nurse-led interventions across chronic conditions [8,10]. Our contribution lies in explicating the specific dimensions of nursing support that participants found valuable: professional expertise, motivational encouragement, and genuine therapeutic relationships. These findings resonate with Watson’s [53] Theory of Human Caring, which emphasizes the healing potential of authentic caring relationships in nursing practice.

The barriers identified by participants, specifically time constraints, physical limitations, environmental factors, and cultural considerations, mirror challenges documented in broader literature on physical activity adherence [7,20]. However, our study situates these barriers within the Saudi Arabian context, highlighting culture-specific dimensions including gender considerations, family dynamics, and climatic challenges that may require tailored intervention strategies.

### 4.3. Contextual Considerations: Physical Activity in Saudi Arabia

The Saudi Arabian context presents unique considerations for physical activity promotion. The extreme heat prevalent for much of the year limits outdoor activity options, underscoring the importance of indoor climate-controlled exercise facilities [54]. Cultural norms regarding gender-appropriate spaces and activities necessitate attention to providing comfortable, culturally sensitive exercise environments for women [14]. The strong family orientation of Saudi society suggests that family-involved interventions may be particularly effective, as reflected in participants’ recommendations.

Saudi Arabia’s Vision 2030 national transformation program includes health promotion objectives that align with expanded nurse-led lifestyle interventions [55]. Our findings provide evidence supporting the integration of nurse-guided physical activity programs into primary healthcare services, consistent with the primary care strengthening emphasis of current health policy.

### 4.4. Implications for Practice and Policy

#### 4.4.1. The Nursing Role: Implications for Practice

Our findings carry significant implications for nursing practice in diabetes care and chronic disease management. The multidimensional support that nurses provide by combining clinical expertise, educational guidance, motivational encouragement, and genuine caring represents a model for holistic nursing intervention that transcends narrowly defined clinical tasks.

The study highlights the importance of nurses’ relational skills alongside their technical competencies. Participants valued not only nurses’ ability to monitor physiological parameters and ensure exercise safety but equally their capacity to listen, encourage, individualize care, and create supportive therapeutic relationships. These relational dimensions align with nursing’s fundamental commitment to person-centered care and should be explicitly cultivated in nursing education and professional development programs [56].

The findings also support expanded nursing roles in health promotion and lifestyle intervention. Nurses’ sustained patient contact, holistic assessment skills, and patient education expertise position them ideally to lead physical activity programs. Healthcare organizations should recognize and support these expanded roles through appropriate staffing, training, and resource allocation.

#### 4.4.2. Implications for Intervention Development

Participants’ recommendations offer practical guidance for refining nurse-guided physical activity programs. Extended program duration or graduated transition phases may support habit formation and maintenance beyond initial intervention periods. Technology integration, facilitated by apps, online platforms or telehealth check-ins, could extend program reach and support sustained engagement. Family involvement in educational components or select exercise sessions may create supportive home environments. Attention to cultural sensitivity, including gender-appropriate facilities and culturally resonant health messaging, remains essential in the Saudi context.

The strong social bonds that participants developed suggest that facilitating peer support networks could enhance program impact and sustainability. Creating opportunities for ongoing group exercise, alumni connections, and peer mentorship may leverage social support mechanisms that participants found valuable.

#### 4.4.3. Healthcare Policy Implications

At the policy level, the findings support investment in nurse-led lifestyle interventions as an integral component of comprehensive diabetes care. Given the escalating burden of T2DM in Saudi Arabia and globally, scalable and sustainable prevention strategies are urgently required. Nurse-guided physical activity programs represent a feasible, acceptable, and effective approach that warrants integration into routine care pathways. Policy attention is also needed to strengthen training infrastructure, as delivering effective physical activity interventions requires competencies in exercise prescription, behavioral counseling, motivational interviewing, and group facilitation, skills that are not consistently emphasized in standard nursing curricula. In addition, reimbursement and healthcare financing policies should recognize and support nurse-led health promotion services, as misaligned incentives that favor curative over preventive care may hinder the sustainable implementation of lifestyle interventions.

### 4.5. Strengths and Limitations

This study possesses several methodological strengths that enhance the credibility and value of its findings. First, the application of reflexive thematic analysis, with systematic and transparent analytic procedures, supports findings that are closely grounded in participant data. Second, the research team comprised experienced qualitative researchers with clinical nursing backgrounds, enabling informed questioning and contextually appropriate interpretation. Third, multiple strategies were employed to enhance trustworthiness, including member checking, peer debriefing, and maintenance of a comprehensive audit trail. Fourth, adherence to COREQ guidelines ensures comprehensive, transparent reporting that enables critical appraisal by readers. Fifth, the study achieved data saturation, with no new themes emerging in later interviews, suggesting adequate sample size for the research aims. Sixth, maximum variation sampling captured diverse perspectives across demographic characteristics and program experiences, enhancing the comprehensiveness of findings.

Several limitations should also be acknowledged. Although participants were recruited from multiple healthcare institutions, the study was conducted within the Saudi healthcare context, which may limit transferability to other settings; however, detailed contextual description supports reader judgment of applicability. Although participants were drawn from both public and private institutions, experiences were not formally compared by institution type, so any influence of institutional setting on participants’ experiences remains unexamined. Participants had all completed the 12-week program, potentially introducing selection bias by excluding non-completers or those with predominantly negative experiences. Interviews were conducted shortly after program completion, capturing immediate rather than long-term experiences. Although interviewers had no pre-existing personal or professional relationship with participants, the healthcare context of recruitment and the recency of program completion mean that social desirability bias, whereby participants may have been inclined to emphasize positive experiences and understate difficulties, cannot be entirely excluded. Coding was concentrated primarily in one researcher, with a second researcher’s independent coding limited to four of sixteen transcripts, which, despite being consistent with reflexive TA’s interpretive stance, represent a narrower base of team-based coding than some readers may expect. No objective clinical outcome data (e.g., HbA1c, blood pressure, or weight measurements) were systematically collected or verified against medical records as part of this qualitative study; all physiological changes described by participants reflect self-reported perceptions rather than confirmed clinical values. Although the sample of 16 was consistent with common recommendations for information power in qualitative descriptive designs [38], a larger or more varied sample might have surfaced additional perspectives, particularly from individuals with lower adherence or less favorable experiences. Finally, because interviews were conducted in Arabic and illustrative quotations translated into English for reporting, some nuance of meaning or expression may not be fully captured in translation, despite efforts to preserve idiomatic content. Finally, the study focused solely on patient perspectives, without incorporating views from nurses, family members, or other stakeholders.

### 4.6. Recommendations

The findings support the integration of nurse-guided physical activity programs into routine diabetes management, emphasizing holistic nursing care that combines clinical expertise, motivational support, and therapeutic relationships. Programs should be individualized, culturally sensitive, and supported by clear safety protocols, with consideration given to extended program duration, technology-enabled delivery, family involvement, and peer support mechanisms to enhance sustainability. Nursing education should incorporate training in physical activity promotion, exercise prescription, behavioral counseling, and motivational interviewing, supported by targeted continuing professional development. At the policy level, healthcare financing, workforce planning, and primary care strengthening initiatives should explicitly support expanded nursing roles in lifestyle intervention delivery. Future research should prioritize longitudinal, mixed-methods, implementation, and cost-effectiveness studies to inform scale-up and policy decision-making.

## 5. Conclusions

This study provides in-depth insights into the experiences of individuals with T2DM participating in a nurse-guided physical activity program. Participants described meaningful physical, psychological, and motivational benefits, underpinned by the supportive and relational role of nurses. These findings reinforce the value of nurse-led lifestyle interventions as patient-centered, effective components of diabetes care. As the global burden of T2DM continues to rise, integrating evidence-based, nurse-guided physical activity programs into routine practice represents a promising strategy to improve clinical outcomes, enhance quality of life, and support the long-term well-being of people living with diabetes.

## Figures and Tables

**Table 1:** Participant characteristics (*n* = 16).

Pseudonym	Gender	Age Category	Duration of T2DM (Years)	BMI Category	Education Level	Employment Status	Treatment Regimen	Session Attendance (%)
P1	Male	45–54	8	Obese	Bachelor’s	Employed	OHA + Insulin	94
P2	Female	45–54	5	Overweight	Secondary	Homemaker	OHA	100
P3	Male	≥55	12	Obese	Master’s	Employed	OHA + Insulin	89
P4	Female	<45	3	Overweight	Bachelor’s	Employed	OHA	97
P5	Male	≥55	18	Overweight	Secondary	Retired	Insulin	83
P6	Female	<45	4	Obese	Diploma	Employed	OHA	100
P7	Male	≥55	10	Obese	Bachelor’s	Employed	OHA + Insulin	86
P8	Female	45–54	7	Overweight	Bachelor’s	Homemaker	OHA	92
P9	Male	45–54	6	Normal	Diploma	Employed	OHA	100
P10	Female	≥55	14	Obese	Secondary	Homemaker	OHA + Insulin	78
P11	Male	<45	2	Overweight	Master’s	Employed	OHA	97
P12	Female	<45	5	Overweight	Bachelor’s	Employed	OHA	94
P13	Male	≥55	15	Obese	Diploma	Retired	Insulin	81
P14	Female	45–54	9	Obese	Secondary	Homemaker	OHA + Insulin	89
P15	Male	45–54	7	Overweight	Bachelor’s	Employed	OHA	92
P16	Female	<45	3	Normal	Master’s	Employed	OHA	100

Note: BMI = Body Mass Index; OHA = Oral Hypoglycemic Agents; T2DM = Type 2 Diabetes Mellitus.

**Table 2:** Summary of themes and subthemes.

Theme	Subtheme	Description
1. Transformative Health Journey	1.1 Perceived physiological improvements	Participants’ observations of changes in blood glucose, blood pressure, weight, and other physical health indicators
	1.2 Enhanced energy and vitality	Increased daily energy levels, reduced fatigue, and improved capacity for physical activities
	1.3 Improved sleep and lifestyle patterns	Positive changes in sleep quality, daily routines, and overall lifestyle habits
2. Psychological Awakening	2.1 Elevated mood and emotional well-being	Improvements in mood, reduced stress, and enhanced emotional balance
	2.2 Strengthened self-efficacy and empowerment	Increased confidence in managing diabetes and performing physical activities
	2.3 Reduced diabetes-related distress	Diminished worry, anxiety, and psychological burden associated with diabetes
3. The Pivotal Role of Nursing Support	3.1 Professional guidance and expertise	Appreciation for nurses’ knowledge, instruction quality, and clinical monitoring
	3.2 Motivational support and encouragement	The importance of nurses’ motivational role in sustaining participation and effort
	3.3 Therapeutic relationship and personalized care	The value of caring relationships and individualized attention
4. Navigating Challenges and Charting Future Directions	4.1 Barriers and obstacles encountered	Challenges including time constraints, physical limitations, and environmental factors
	4.2 Facilitators of success	Factors enabling program participation and adherence
	4.3 Recommendations for enhancement	Participants’ suggestions for improving future programs

## Data Availability

The data presented in this study are available on request from the corresponding author. The data are not publicly available due to privacy and ethical restrictions.

## Supplementary Materials

The following supporting information can be downloaded at https://www.mdpi.com/article/10.3390/healthcare14172756/s1: Supplementary File S1: COREQ checklist; Supplementary File S2: Semi-structured interview guide.
