# Supplementary material for: Patients’ Experiences of a Nurse-Guided Physical Activity Program for Type 2 Diabetes and Its Perceived Impact on Health and Quality of Life: A Qualitative Descriptive Study
Source: Healthcare (Basel). 2026 Aug 31;14(17):2756. doi: 10.3390/healthcare14172756 (PMC13565185; doi:10.3390/healthcare14172756)
Supplement: Supplementary file 1 [file healthcare-14-02756-s001.zip › healthcare-4530074-supplementary.pdf]

Supplementary Materials

Supplementary File S1. COREQ Checklist

Consolidated Criteria for Reporting Qualitative Research (COREQ): 32-Item Checklist

No.	Item	Guide Questions/Description	Reported on
Domain 1: Research Team and Reflexivity			
Personal Characteristics			
1	Interviewer/facilitator	Which author/s conducted the interview or focus group?	Methods section
2	Credentials	What were the researcher's credentials? e.g., PhD, MD	Title page
3	Occupation	What was their occupation at the time of the study?	Title page
4	Gender	Was the researcher male or female?	Title page/ Methods
5	Experience and training	What experience or training did the researcher have?	Methods section (Researcher Reflexivity)
Relationship with Participants			
6	Relationship established	Was a relationship established prior to study commencement?	Methods section
7	Participant knowledge of the interviewer	What did the participants know about the researcher? e.g., personal goals, reasons for doing the research	Methods section (Ethical considerations)
8	Interviewer characteristics	What characteristics were reported about the interviewer/facilitator? e.g., bias, assumptions, reasons and interests in the research topic	Methods section (Researcher Reflexivity)
Domain 2: Study Design			
Theoretical Framework			
9	Methodological orientation and theory	What methodological orientation was stated to underpin the study? e.g., grounded theory, discourse analysis, ethnography, phenomenology, content analysis	Methods section (Study Design)
Participant Selection			
10	Sampling	How were participants selected? e.g., purposive, convenience, consecutive, snowball	Methods section (Participants and Sampling)
11	Method of approach	How were participants approached? e.g., face-to-face, telephone, mail, email	Methods section
12	Sample size	How many participants were in the study?	Findings section (Table 1)

13	Non-participation	How many people refused to participate or dropped out? Reasons?	Methods section
Setting			
14	Setting of data collection	Where was the data collected? e.g., home, clinic, workplace	Methods section (Data Collection)
15	Presence of non-participants	Was anyone else present besides the participants and researchers?	Methods section
16	Description of sample	What are the important characteristics of the sample? e.g., demographic data, date	Findings section (Table 1)
Data Collection			
17	Interview guide	Were questions, prompts, guides provided by the authors? Was it pilot tested?	Methods section; Supplementary File 3
18	Repeat interviews	Were repeat interviews carried out? If yes, how many?	Methods section
19	Audio/visual recording	Did the research use audio or visual recording to collect the data?	Methods section (Data Collection)
20	Field notes	Were field notes made during and/or after the interview or focus group?	Methods section (Data Collection)
21	Duration	What was the duration of the interviews or focus group?	Methods section (Data Collection)
22	Data saturation	Was data saturation discussed?	Methods section (Participants and Sampling)
23	Transcripts returned	Were transcripts returned to participants for comment and/or correction?	Methods section (Trustworthiness)
Domain 3: Analysis and Findings			
Data Analysis			
24	Number of data coders	How many data coders coded the data?	Methods section (Data Analysis)
25	Description of the coding tree	Did authors provide a description of the coding tree?	Supplementary File 4
26	Derivation of themes	Were themes identified in advance or derived from the data?	Methods section (Data Analysis)
27	Software	What software, if applicable, was used to manage the data?	Methods section (Data Analysis)
28	Participant checking	Did participants provide feedback on the findings?	Methods section (Trustworthiness)
Reporting			
29	Quotations presented	Were participant quotations presented to illustrate the themes/findings? Was each quotation identified? e.g., participant number	Findings section
30	Data and findings consistent	Was there consistency between the data presented and the findings?	Findings section
31	Clarity of major themes	Were major themes clearly presented in the findings?	Findings section; Table 2
32	Clarity of minor themes	Is there a description of diverse cases or discussion of minor themes?	Findings section

Supplementary File S2. Semi-Structured Interview Guide

Introduction

Interviewer reads to participant:

"Thank you for agreeing to participate in this interview. I am a researcher from King Saud University. We are conducting this study to understand patients' experiences with the nurse-guided physical activity program you recently completed. Your insights will help us improve future programs and better support patients with diabetes.

This interview will take approximately 45-60 minutes. I would like to record our conversation to ensure accuracy is that acceptable to you? Your responses will be kept confidential, and your name will not appear in any reports. You may choose not to answer any question and may stop the interview at any time.

Do you have any questions before we begin?"

Part 1: Overall Program Experience (Questions 1-5)

Q1. Opening Question: "To begin, can you tell me about your overall experience participating in the nurse-guided physical activity program?"

- *Probes:* What was it like for you? How would you describe the experience to someone who hasn't participated?

Q2. First Impressions: "What were your first impressions when you started the program? Did these change over time?"

- *Probes:* What did you expect? Was it different from what you expected?

Q3. Commitment Level: "How would you describe your level of commitment throughout the twelve weeks? Did it fluctuate?"

- *Probes:* What helped you stay committed? Were there times you considered stopping?

Q4. Program Components: "Which aspects of the program did you find most beneficial? Which were least helpful?"

- *Probes:* Specific exercises? Education components? Group vs. individual activities?

Q5. Emotional Response: "What emotions did you experience during your participation in the program?"

- *Probes:* Before sessions? During? After? Over the 12 weeks?

Part 2: Perceived Health Changes (Questions 6-9)

Q6. Physical Health Changes: "Have you noticed any changes in your physical health since starting the program?"

- *Probes:* Blood sugar levels? Blood pressure? Weight? Energy levels? Sleep?

Q7. Diabetes Management: "How has participation in the program affected how you manage your diabetes day-to-day?"

- *Probes:* Medication? Monitoring? Healthcare visits? Diet?

Q8. Physical Capabilities: "Have you noticed changes in your physical abilities or what your body can do?"

- *Probes:* Stamina? Strength? Flexibility? Ability to perform daily activities?

Q9. Health Monitoring: "What have your clinical measurements (blood tests, blood pressure, etc.) shown since you started the program?"

- *Probes:* What do these numbers mean to you?

Part 3: Quality of Life Impact (Questions 10-13)

Q10. Overall Quality of Life: "How would you describe the impact of this program on your overall quality of life?"

- *Probes:* What does "quality of life" mean to you? In what ways has it changed?

Q11. Psychological Well-being: "Has participating in the program affected your mood, stress levels, or psychological well-being?"

- *Probes:* Anxiety about diabetes? Depression? Self-confidence? Sense of control?

Q12. Social Life: "Has the program affected your relationships or social life in any way?"

- *Probes:* Family relationships? Friendships? Social activities?

Q13. Daily Life: "How has your participation in the program affected your daily routine and activities?"

- *Probes:* Work? Household responsibilities? Hobbies? Leisure time?

Part 4: Role of Nursing Support (Questions 14-16)

Q14. Nurse Guidance: "How would you describe the support and guidance you received from the nurses during the program?"

- *Probes:* What specifically did they do that was helpful? Could anything have been done differently?

Q15. Importance of Nursing Role: "In your view, how important was having nurses lead and supervise the program?"

- *Probes:* Would the experience have been different without nursing involvement? How?

Q16. Therapeutic Relationship: "How would you describe your relationship with the nursing staff during the program?"

- *Probes:* Trust? Communication? Personalization of care?

Part 5: Barriers and Facilitators (Questions 17-18)

Q17. Challenges: "What challenges or difficulties did you face while participating in the program?"

- *Probes:* Physical challenges? Time constraints? Family or work responsibilities? Motivational challenges?

Q18. Facilitators: "What factors helped you participate in and complete the program?"

- *Probes:* Personal factors? Support from others? Program features? External factors?

Part 6: Future Directions (Questions 19-20)

Q19. Recommendations: "If you could change or improve anything about the program, what would you suggest?"

- *Probes:* Content? Format? Duration? Frequency? Support offered?

Q20. Sustainability: "Now that the program has ended, what are your plans for maintaining physical activity?"

- *Probes:* Confidence to continue? Barriers anticipated? Resources needed?

Closing (3-5 minutes)

"Is there anything else you would like to share about your experience with the program that we haven't discussed?"

Thank you very much for your time and for sharing your experiences. Your insights are invaluable and will help us improve future programs. If you have any questions or wish to add anything later, please feel free to contact me.
